# Light Levels Affect Carbon Utilisation in Tropical Seagrass under Ocean Acidification

**DOI:** 10.1371/journal.pone.0150352

**Published:** 2016-03-03

**Authors:** Yan X. Ow, Sven Uthicke, Catherine J. Collier

**Affiliations:** 1 College of Marine and Environmental Science, James Cook University, Townsville, Queensland, Australia; 2 Australian Institute of Marine Science, Townville, Queensland, Australia; 3 Experimental Marine Ecology Laboratory, Department of Biological Sciences, National University of Singapore, Singapore, Singapore; 4 Centre for Tropical Water & Aquatic Ecosystem Research (TropWATER), James Cook University, Cairns, Queensland, Australia; University of Hong Kong, HONG KONG

## Abstract

Under future ocean acidification (OA), increased availability of dissolved inorganic carbon (DIC) in seawater may enhance seagrass productivity. However, the ability to utilise additional DIC could be regulated by light availability, often reduced through land runoff. To test this, two tropical seagrass species, *Cymodocea serrulata* and *Halodule uninervis* were exposed to two DIC concentrations (447 μatm and 1077 μatm *p*CO_2_), and three light treatments (35, 100, 380 μmol m^-2^ s^-1^) for two weeks. DIC uptake mechanisms were separately examined by measuring net photosynthetic rates while subjecting *C*. *serrulata* and *H*. *uninervis* to changes in light and addition of bicarbonate (HCO_3_^-^) use inhibitors (carbonic anhydrase inhibitor, acetazolamide) and TRIS buffer (pH 8.0). We observed a strong dependence on energy driven H^+^-HCO_3_^-^ co-transport (TRIS, which disrupts H^+^ extrusion) in *C*. *serrulata* under all light levels, indicating greater CO_2_ dependence in low light. This was confirmed when, after two weeks exposure, DIC enrichment stimulated maximum photosynthetic rates (P_max_) and efficiency (α) more in *C*. *serrulata* grown under lower light levels (36–60% increase) than for those in high light (4% increase). However, *C*. *serrulata* growth increased with both DIC enrichment and light levels. Growth, NPP and photosynthetic responses in *H*. *uninervis* increased with higher light treatments and were independent of DIC availability. Furthermore, *H*. *uninervis* was found to be more flexible in HCO_3_^-^ uptake pathways. Here, light availability influenced productivity responses to DIC enrichment, via both carbon fixation and acquisition processes, highlighting the role of water quality in future responses to OA.

## Introduction

Seagrass meadows are highly productive habitats that offer a wide range of ecologically and economically valuable ecosystem services [1]. Especially important is their ability to capture and convert light energy into organic matter, which then become available to other trophic levels [2, 3]. This primary production is strongly determined by the amount of light available in the aquatic environment [4]. When coastal water quality declines, eutrophication and high turbidity in the water column reduce the light available to these primary producers [5]. Chronic light limitation had been shown to lower seagrass productivity and contribute to the accelerating areal loss of these habitats [6–8].

Seagrass productivity may also be limited by the present day seawater composition of dissolved inorganic carbon (DIC) [9, 10]. However, with the rising emissions of carbon dioxide (CO_2_) from anthropogenic activities being continually absorbed by the ocean, there is a shift in the relative proportion of each DIC species (HCO_3_^-^, CO_3_^2-^, CO_2_) in seawater [11, 12] as the pH is reduced (Ocean acidification, or OA). Importantly, the proportion of CO_2_ will have the greatest percent increase (>250%, compared to 24% for HCO_3_^-^) among the DIC constituents under the projected pH decrease by 2100 [13]. Studies had indicated that greater availability of DIC under future OA conditions may benefit seagrasses [13–15]. An increase in photosynthesis and growth following exposure to increased DIC in both tropical and temperate species was previously demonstrated [16–22]. Moreover, since tropical and temperate seagrasses displayed a higher photosynthetic affinity for CO_2_ than HCO_3_^-^, an increased availability of CO_2_ can further boost productivity [23, 24]. At natural CO_2_ vent sites, growth and areal cover of seagrasses were observed to be much greater than at adjacent non-CO_2_ enriched sites [15, 25]. Hence, given sufficient light and nutrient availability, seagrasses could utilise the extra provisioning of DIC to enhance productivity [9, 22, 23, 26].

Seagrasses can use bicarbonate (HCO_3_^-^), the dominant DIC species, for photosynthesis [27]. Bicarbonate utilization involves the dehydration and transport of HCO_3_^-^ into the plant cells [27, 28]. Various HCO_3_^-^ utilization pathways that have been proposed involved the enzyme carbonic anhydrase (CA) and the presence of acidic zones maintained by proton (H^+^) gradients [26, 28, 29]. Extracellular CA dehydrates HCO_3_^-^ to CO_2_, allowing CO_2_ to diffuse into the cell. Another uptake pathway involves H^+^ extrusion-driven co-transport of H^+^ and HCO_3_^-^ [30]. In general, active extrusion of H^+^ into localized regions of the leaf boundary layer (acidic zones) for HCO_3_^-^ utilisation is energetically costly compared to passive CO_2_ uptake, and thus could be limited by low light levels [22].

Light availability can influence the ability of seagrasses to exploit enriched DIC conditions for photosynthesis. Fluctuating light availability [31–33] could vary the carbon demand for the downstream carbon fixation cycle. On the other hand, elevated DIC (with CO_2_ as substrate) increased photosynthetic efficiency and reduced light requirements of seagrasses [19, 22]. So far, the interaction of light and DIC availability on seagrass photosynthesis has only been explored in a few studies [16, 19, 34–36]. Productivity responses in seagrasses to DIC enrichment under low light conditions had been variable, ranging from no change in growth rates in *Zostera marina* [16], to increased growth in *Amphibolis antarctica* [35] and enhanced photosynthesis in *Halophila ovalis*, *Cymodocea serrulata* [34] and *Thalassia hemprichii* [36]. Studies demonstrating positive effects of DIC enrichment under low light [34, 35] suggested that seagrass growing under low light may benefit more from DIC enrichment, through a lowered epiphyte load and/or reduced energetic demand from HCO_3_^-^ use with an increase in CO_2_ supply. However, it has to be noted that some of these studies did not directly measure light levels, instead using water depth [34] and epiphyte cover [36] as proxies for light reduction. It is unclear if this DIC limitation stemmed directly from a reduced HCO_3_^-^ utilization due to low light availability, or was compounded with reduced mixing or competitive DIC uptake by epiphytic algae.

This study aimed to examine the effects of light availability on carbon utilisation in two tropical seagrasses, *C*. *serrulata* and *Halodule uninervis*. Growth, net primary productivity and photosynthetic responses to DIC enrichment under different light treatment levels were characterised in a two-week aquaria experiment. DIC levels were chosen to represent present day and end-of-century (CO_2_ ~ 1000ppm) emission scenarios (RCP8.5) [37]. It was hypothesized that while DIC enrichment (7.5% increase relative to ambient seawater) will increase photosynthesis and growth of seagrasses, the extent of increase will be greater under lower light levels. To further test the influence of light availability on HCO_3_^-^ use in tropical seagrasses, HCO_3_^-^ utilization mechanisms were investigated using CA and H^+^ gradient inhibitors (CA-mediated and/or H^+^ co-transport mediated) under contrasting light levels [23, 38]. Energetic demand from using HCO_3_^-^ may make it unfavourable as a photosynthetic substrate under limiting light levels. Hence the hypothesis was that under limiting light availability, the ability to use of HCO_3_^-^ as a carbon substrate would be reduced [39, 40].

## Materials and Methods

### Experimental species

Two common tropical seagrass species, *Cymodocea serrulata* and *Halodule uninervis*, were collected from an intertidal meadow at Cockle Bay, Magnetic Island, northern Great Barrier Reef (19°10.88’S, 146°50.63’E). Photosynthetically active radiation (PAR) was measured at Cockle Bay using planar irradiance collectors (Odyssey Photosynthetic Irradiance Recording System, Dataflow Systems Pty Ltd, New Zealand) installed at seagrass canopy height from 2009 to 2014. The mean integrated daily irradiance at Cockle Bay from September 2009 to July 2014 was 14.9 mol m^-2^ d^-1^. Calculated over the light period, the average PAR at this site was 385 μmol m^-2^ s^-1^, the average maximum PAR was 961 μmol m^-2^ s^-1^, while the median PAR was 196 μmol m^-2^ s^-1^ [41]. Variation in light levels between the wet and dry season was low (S1 Fig). Average water temperature at this site (2005 to 2012) was around 26°C [41]. Cores of *H*. *uninervis* and sediment were collected as intact plugs and *C*. *serrulata* as intact horizontal rhizomes [21], first in July 2012 for the DIC and light aquaria study, and later in May 2014 for the inhibitors study (detailed below). On both occasions, seagrasses were collected under a limited impact research permit (MTB41), which was assessed and issued by the Department of Employment, Economic Development and Innovation (Fisheries Queensland Code MP05) for the removal of marine plants from the Great Barrier Reef Marine Park. The potted seagrasses were kept in an outdoor flow-through aquarium (1000 L) with filtered seawater (5 μm) for a three-week acclimation period. Maximum light level in the outdoor aquarium was 350 μmol m^-2^ s^-1^ with seawater temperature of 23–25°C and salinity at 35–36 (PSS-78).

### Light and DIC effects on photosynthetic and growth response

#### Experimental set-up

The seagrasses were exposed to three different light levels (35, 100 and 380 μmol m^-2^ s^-1^) and two seawater DIC concentrations (high vs control; Table 1) in an indoor flow-through system over two weeks. Two weeks exposure were shown to be sufficient for inducing photosynthetic changes in the two species in a previous experiment [21]. The three light levels chosen for the experiment (35, 100 and 380 μmol m^-2^ s^-1^) provided 1.5, 4.3 and 16.4 mol m^-2^ d^-1^ of light per day over a 12:12 h light:dark cycle. The two lower light levels represented low light conditions that were less common, but still ecologically relevant at the source meadow. The highest light level (380 μmol m^-2^ s^-1^) provided an integrated daily irradiance which was similar to that most commonly encountered at Cockle Bay (12–14 mol m^-2^ d^-1^). The experiment was conducted at the Australian Institute of Marine Sciences, Townsville. Eighteen glass aquaria (working volume 18 litres) with three replicates for each treatment were supplied with fresh filtered (5 μm) seawater from header tanks. One pot of each seagrass species was placed into each aquarium. Leaves were cleaned of epiphytes every two days by gentle rubbing between fingers. Light and CO_2_ levels were randomly assigned to each aquarium. LED lights mounted over the top of each aquarium provided illumination to cover the full sunlight spectrum (Aqua Illumination LED, USA). The LED lights were set to provide 380 μmol m^-2^ s^-1^. Irradiance was determined using a planar irradiance meter (LICOR, USA). Light reduction was achieved by placing neutral density shade-cloth over individual aquaria. DIC concentrations were manipulated by means of feedback-regulated CO_2_ input (AquaMedic, Germany) into the header tanks, as described in [42]. In all the header and aquarium tanks, diffusers and pumps were installed to ensure thorough mixing of DIC enriched water. Additional pH and temperature measurements were taken manually (pH probe: Eutech, USA; console: Oakton, USA) and pH levels calibrated to TRIS seawater standards (Batch 10, Supplied by A. Dixon, Scripps Institute of Oceanography). Salinity was measured with a handheld refractometer. Every four days, water samples were taken from each aquarium and analysed for dissolved inorganic carbon (DIC) and total alkalinity (AT) concentrations using a Vindta 3C analyser. Measured values of DIC, AT, temperature and salinity were used to calculate carbonate system parameters in USGS CO2calc software [43]. Water samples for inorganic nutrient (NH_4_^+^ and NO_3_^-^) measurements were collected in duplicate, every five days, from each aquarium, filtered (0.45 μm) and analysed [44].

**Table 1:** Measured and calculated carbonate system parameters for high DIC and control treatments.

	Measured parameters					Calculated parameters					Nutrient concentrations		
DIC treatment	DIC (μmol kg^-1^SW)	pH [NBS]	AT (μmol kg^-1^ SW)	Temp (°C)	Salinity (psu)	pH [NBS]	pCO_2_ (μatm)	CO_2_ (μmol kg^-1^SW)	HCO_3_^-^ (μmol kg^-1^ SW)	CO_3_^2-^ (μmol kg^-1^ SW)	NH_4_^+^ (μmol/L)	NO_3_^-^ (μmol/L)	NO_2_^-^ (μmol/L)
High	2215 (16.1)	7.85 (0.1)	2327 (8.4)	23.8 (0.3)	36.4 (0.7)	7.82 (0.04)	1077 (104.1)	31.7 (3.1)	2083 (20.6)	101 (8.2)	0.12 (0.01)	1.35 (0.11)	0.07 (0.01)
Control	2063 (9.8)	8.26 (0.1)	2327 (10.0)	23.9 (0.3)	36.4 (0.8)	8.15 (0.02)	447 (23.1)	13.1 (0.7)	1858 (14.1)	192 (7.1)	0.13 (0.01)	1.26 (0.11)	0.07 (0.01)

Temperature and pH readings in the aquaria tanks were measured with a hand held pH probe calibrated on the NBS scale. Dissolved inorganic carbon (DIC) and total alkalinity (AT) concentrations were measured from water samples taken every four days from each aquaria tank. Carbonate system parameters were calculated using USGS CO2calc software [43]. Average values and S.D. (in brackets) were given.

#### Growth measurements

Growth rates of seagrass shoots in the experiment were measured following [45]. At the start, all shoots were marked at the top of the bundle sheath with a needle. After two weeks of growth, the shoots were harvested; new tissue growth was excised and dried at 60°C for 48 hours before weighing. Biomass of new tissue growth was normalised to the total above-ground biomass in each pot to calculate relative growth rates (RGR, g g^-1^ DW day^-1^).

#### Photosynthesis vs irradiance curves

To characterise photosynthetic parameters, dark respiration and photosynthetic rates over a light range were measured to construct photosynthesis vs irradiance (P-E) curves two weeks after the initiation of the experiment. Oxygen consumption and evolution rates of seagrass leaves in seawater from their respective treatment aquaria (DIC enriched: 1077 ± 104 μatm; control: 447 ± 23 μatm) were monitored using optical oxygen sensors (Sensor spots-PSt3, PreSens) and a fibre-optic oxygen meter (PreSens Oxy 4) in 200 mL volume incubation chambers [21]. The chambers were incubated at 25°C water temperature in a flow-through water bath system (Lauda, Ecoline RE 106). Magnetic stirrers (2 cm × 0.8 cm, 128 revolutions per minute) provided even mixing in each chamber. One mature epiphyte-free (rank 2) leaf was held upright in the chamber to mimic natural orientation. Respiration was measured over a 20-min period in the dark. Photosynthetic rates were measured on the same leaf over a series of light steps (10, 20, 35, 70, 100, 200, 380, 520, 600 μmol m^-2^ s^-1^), with each light step lasting 25 min. Adjustable LED lights were used to provide the different light intensities (Aqua Illumination LED). Light spectra of LED lights are provided in S2 Fig. Incubation media in chambers were replaced with fresh filtered seawater from respective aquaria prior to measurements at these light steps: 35, 100, 380 and 600 μmol m^-2^ s^-1^. Initial trials showed that low photosynthetic rates at low light levels, and short incubation times between replacements of incubation media at higher light levels reduced the possibility of DIC limitation during incubations (S1 Table). Dark respiration and photosynthetic rates were derived by fitting a linear regression to the logged oxygen concentration data in the each chamber. Only steady-state measurements of respiration and photosynthesis (obtained after ~5 min) were used for regressions. After incubation, leaves were dried (60°C, 48 h) and weighed. Rates were normalised to the dry weight of the leaf. Each optical oxygen sensor was calibrated prior to use according to method described in [46].

Net primary productivity (NPP) was taken to be the photosynthetic rate measured at the respective experimental light levels (35, 100 and 380 μmol m^-2^ s^-1^). To derive photosynthetic parameters, photosynthetic rates were fitted to the adapted hyperbolic tangent model of [47]. The model describes the linear increase (photosynthetic efficiency, α, mg O_2_ μmol^-1^ photons) in photosynthetic rates with irradiance, up until the saturating irradiance (E_k_, μmol m^-2^ s^-1^) where photosynthesis plateaus at the maximum rate (P_max_, mg O_2_ g^-1^ DW h^-1^). Compensation irradiance (E_c_, μmol m^-2^ s^-1^) is the light level when photosynthetic rate is equal to respiration rate.

### Effect of light levels on HCO_3_^-^ uptake

To assess the effect of light on HCO_3_^-^ utilization, net photosynthesis under specific inhibition of HCO_3_^-^ uptake mechanisms was measured under two contrasting light levels. The light levels represented limiting (40 μmol m^-2^ s^-1^) and saturating (600 μmol m^-2^ s^-1^) light as determined from the previous experiment where the derived saturating light (E_k_) was 100 and 98 μmol m^-2^ s^-1^ for *C*. *serrulata* and *H*. *uninervis* respectively. Therefore, the limiting light level was well below E_k_, and the saturating light level was above E_k_ for both species.

Bicarbonate utilization pathways can be elucidated by the effect of specific inhibitors on photosynthetic rates, as described in [28]. One pathway involves the membrane-bound extracellular CA dehydrating HCO_3_^-^ into CO_2_, which then diffuses into the cell. Addition of acetalzolamide (AZ) inhibits extracellular CA activity [28]. Another possible pathway for HCO_3_^-^ utilization consists of localised active H^+^ extrusion to create H^+^ gradients that facilitate the inward co-transport of H^+^ and HCO_3_^-^. Addition of a buffer, e.g. Tris (hydroxymethyl) aminomethane (TRIS), dissipates the H^+^ gradient as the buffer reacts with the extruded H^+^, thus altering photosynthetic rates [27]. A third HCO_3_^-^ utilization pathway consists of extracellular CA-mediated HCO_3_^-^ conversion to CO_2_ in acidic zones created at H^+^ extrusion sites. The acidic zones help concentrate CO_2_ and facilitate diffusion into the cells. Strong inhibition of photosynthetic rates by the combined addition of buffer and AZ indicates a strong dependence on this pathway [29, 48]. Thus, we used AZ, TRIS and their combination in the experiments described below.

Net photosynthesis was determined by measuring rates of oxygen evolution in 60mL incubation chambers, similar to the procedure described above. During measurements, incubation media (fresh filtered seawater (pH 8.0) with or without inhibitor) in each chamber were stirred and maintained at 25°C. Mature and non-epiphytised leaves from non-connected shoots collected fresh from the field, were used for the measurements. Prior to the addition of the inhibitor(s), control rates of dark respiration and photosynthesis for each leaf were measured in fresh filtered seawater (pH 8.0). Dark respiration rates of the leaves were measured over 20 min, before the lights were switched on to provide 40 μmol m^-2^ s^-1^ of light. Net photosynthesis was then measured for 30 min. Subsequently, light levels were increased to 600 μmol m^-2^ s^-1^ and photosynthetic rates of the same leaves were measured. After the control measurements, incubation media for all chambers were replaced with fresh filtered seawater mixed with inhibitor solutions (AZ, TRIS or AZ+TRIS). The second set of incubations used the same leaves and followed the same sequence of measurements (in dark, at limiting light, then at saturating light). Oxygen production and consumption rates were calculated and normalised to leaf dry weight as above. For this experiment, net photosynthetic rates to TRIS and/or AZ were expressed as percentages of the control net photosynthetic rates.

Three inhibitor solutions were used: 1) 0.1 mM AZ, 2) 45 mM TRIS, and 3) combination of 0.1 mM AZ and 45 mM TRIS [38]. A stock solution of 20 mM AZ was prepared by dissolving the powder in 50 mM sodium hydroxide (NaOH). An aliquot of 350μl AZ stock solution was added to each individual chamber to achieve a final concentration of 0.1 mM. TRIS was prepared as 1 M stock solution and adjusted to pH 8.0. The buffer yielded pH 8.0 when mixed with seawater to a final concentration of 45 mM. The final TRIS concentration used here, although was lower than that commonly found in literature [22, 38], was sufficient in eliciting a reduction in net photosynthetic rates.

### Statistical analyses

To evaluate effects of DIC and light for each species, growth, net primary productivity and photosynthetic parameters were analysed using univariate two-factor ANOVA to test for significance in fixed effects of light (three levels) and DIC (two levels) and their interaction. Data were checked for homogeneity of variance using Levene's test and for normality using Q-Q plots. Where the assumptions of ANOVA were violated, data were square root transformed. However when the assumptions were still not met, as observed in the E_c_ and α data for *H*. *uninervis*, the alpha-value was lowered to 0.01 to minimize the risk of a Type I error [49]. In case of significant light effects, Tukey’s HSD was carried out to test which of the treatment levels were significantly different from each other. Similarly, Tukey’s HSD was used to test for significance between selected groups in case of significant light and DIC interactions.

To determine if the addition of AZ and/or TRIS significantly affects net productivity of each seagrass species in fresh seawater (control), raw net productivity data were compared using paired t-tests. Further, to test if light levels affect HCO_3_^-^ utilization, paired t-tests were used to compare the change in percentage net photosynthesis between the two light levels for each inhibitor type. Arcsine transformation was not performed on the the percentage net photosynthesis data, since the data exceeded the range of 0 to 100. Instead, percentage net photosynthesis responses were square root transformed prior to analysis to meet the assumption of normality for the test. All statistical analyses were conducted using R statistical software [50].

## Results

### Light and DIC effects on photosynthetic and growth response

#### Experimental parameters

Water temperature and salinity in the experimental aquaria did not vary considerably throughout the experiment (average ± S.D.: 23.9 ± 0.3°C and 35.9 ± 0.5 (PSS-78)). Manipulation of the CO_2_ system resulted in a 140% increase in CO_2_ concentration, a 10% increase in HCO_3_^-^ concentration, and a corresponding decrease of ~0.3 units in pH, compared to present day control conditions (Table 1). Carbonate system parameters of the DIC treatments remained in the target range. Nutrient concentrations were similar between aquarium tanks; with an average (± S.D.) water column ammonium concentration of 0.12 ± 0.03 μM and nitrate concentration of 1.30 ± 0.55 μM.

#### Productivity responses to light and DIC enrichment

**Fig 1:**
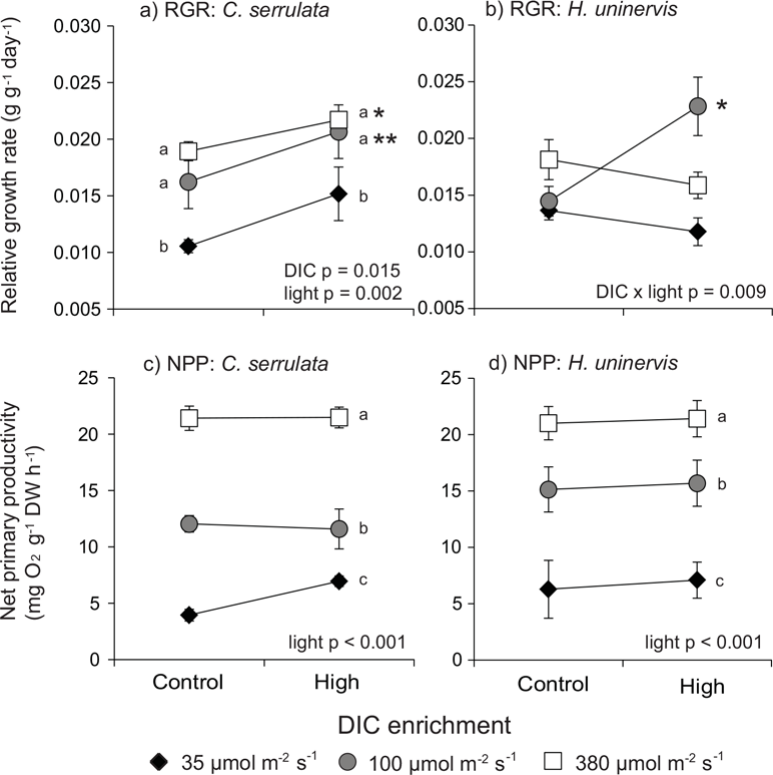
Comparison of relative growth rates and net primary productivity of *C*. *serrulata* and *H*. *uninervis* after two weeks exposure to light and DIC. (a-b) RGR- relative growth rate, (c-d) NPP–net primary productivity. Letters indicate significant differences between light treatments based on Tukey’s HSD test; asterisks indicate significant differences between DIC treatments based on ANOVA results where there is no interaction between light and DIC, or based on Tukey’s HSD test if an interaction was detected (* p < 0.05; ** p < 0.01). Control DIC = 2063 μM; high DIC = 2215 μM. Means (± S.E.) are given (n = 3).

For *C*. *serrulata*, DIC enrichment and light availability both increased relative growth rates (RGR) significantly (ANOVA: DIC p = 0.015; light p = 0.002) (Table 2; Fig 1A). When light levels were at 100 μmol m^-2^ s^-1^ and 380 μmol m^-2^ s^-1^, RGRs increased by 20% and 26% respectively, relative to that at 35 μmol m^-2^ s^-1^ (Tukey HSD: 100 μmol m^-2^ s^-1^ p = 0.015; 380 μmol m^-2^ s^-1^: p = 0.002). DIC enrichment raised RGR by 13%. There was no significant interaction between light and DIC enrichment on RGR in *C*. *serrulata*. In *H*. *uninervis*, effect of DIC enrichment on RGR varied with light levels (ANOVA: p = 0.009) (Table 2; Fig 1B). DIC enrichment increased RGR only for seagrass growing at 100 μmol m^-2^ s^-1^ (Tukey HSD: p = 0.027), but not at lower (Tukey HSD: p = 0.955) or higher (ANOVA: p = 0.905) light levels.

**Table 2:** Two-way ANOVA results.

		*Cymodocea serrulata*				*Halodule uninervis*			
		df	MS	F	P	df	MS	F	p
RGR	DIC	1	1.07 × 10^−3^	8.115	**0.015**	1	8.94 × 10^−6^	1.189	0.297
	Light	2	1.43 × 10^−3^	10.793	**0.002**	2	5.63 × 10^−5^	7.488	**0.008**
	DIC x light	2	3.84 × 10^−5^	0.290	0.753	2	5.45 × 10^−5^	7.238	**0.009**
NPP	DIC	1	3.400	1.116	0.312	1	1.600	0.142	0.713
	Light	2	389.100	126.762	**8.52 × 10**^**−9**^	2	320.800	29.000	**2.54 × 10**^**−5**^
	DIC x light	2	5.200	1.705	0.223	2	0.100	0.006	0.994
Dark	DIC	1	18.924	2.033	0.179	1	0.002	0.003	0.957
respiration	Light	2	23.395	2.513	0.123	2	2.584	4.697	**0.031**
	DIC x light	2	0.163	0.018	0.983	2	0.545	0.991	0.400
P_max_	DIC	1	60.320	27.800	**1.97 × 10**^**−4**^	1	4.320	0.514	0.487
	Light	2	190.160	87.630	**6.92 × 10**^**−8**^	2	126.060	14.992	**5.45 × 10**^**−4**^
	DIC x light	2	8.500	3.920	**0.049**	2	2.550	0.304	0.744
E_k_	DIC	1	8181	39.080	**4.25 × 10**^**−5**^	1	150	0.293	0.598
	Light	2	3475	16.597	**3.50 × 10**^**−4**^	2	3668	7.149	**0.009**
	DIC x light	2	1132	5.409	**0.021**	2	2547	4.964	**0.027**
E_c_	DIC	1	21.500	0.204	0.660	1	1.315	0.789	0.392
	Light	2	534.500	5.053	**0.026**	2	8.073	4.843	0.029
	DIC x light	2	29.200	0.276	0.763	2	8.977	5.385	0.021
α	DIC	1	1.53 × 10^−2^	5.513	**0.037**	1	1.14 × 10^−2^	2.413	0.146
	Light	2	2.24 × 10^−4^	0.081	0.923	2	2.22 × 10^−3^	0.469	0.637
	DIC x light	2	1.91 × 10^−3^	0.686	0.522	2	0.024	5.049	0.026

All parameters were analysed with DIC and light treatments as fixed factors. N = 3. Significant p-values are in bold. Prior analysis, square-root transformation had been applied to RGR for *Cymodocea serrulata*, and to E_c_, α and respiration for *Halodule uninervis*. RGR- relative growth rate, P_max_- maximum photosynthetic rate, E_k_- saturating irradiance, E_c_- compensation irradiance, α- photosynthetic efficiency.

In *C*. *serrulata*, net primary productivity (NPP) was significantly influenced by light levels (ANOVA: p < 0.001) (Table 2; Fig 1C). DIC enrichment did not significantly raise NPP (ANOVA: p = 0.312) (Table 2). NPP in *C*. *serrulata* increased with light, by 12% (at 100 μmol m^-2^ s^-1^) and 29% (at 380 μmol m^-2^ s^-1^) (Tukey HSD: 100 μmol m^-2^ s^-1^: p < 0.001; 380 μmol m^-2^ s^-1^: p < 0.001). Similarly for *H*. *uninervis*, NPP increased significantly with light but not with DIC enrichment (ANOVA: light p < 0.001; DIC p = 0.713) (Table 2; Fig 1D). NPP increased by 13 to 22% in *H*. *uninervis* with light (Tukey HSD: 100 μmol m^-2^ s^-1^: p = 0.002; 380 μmol m^-2^s^-1^: p < 0.001). Dark respiration rates did not vary with light treatments or DIC in *C*. *serrulata* (Table 2). In *H*. *uninervis*, dark respiration rates responded to light treatments only (ANOVA: p = 0.031). Dark respiration rates increased by 67% at 380 μmol m^-2^ s^-1^ relative to at 35 μmol m^-2^ s^-1^ (Tukey HSD: p = 0.032).

#### Photosynthetic-irradiance (P-E) curves

The adapted hyperbolic tangent model provided a good fit for all P-E curves (R^2^ > 0.85; p < 0.050). Photosynthetic rates increased linearly (initial slope, α) with irradiance before plateauing off at the maximum photosynthetic rate (P_max_) above saturating irradiance (E_k_).

**Fig 2:**
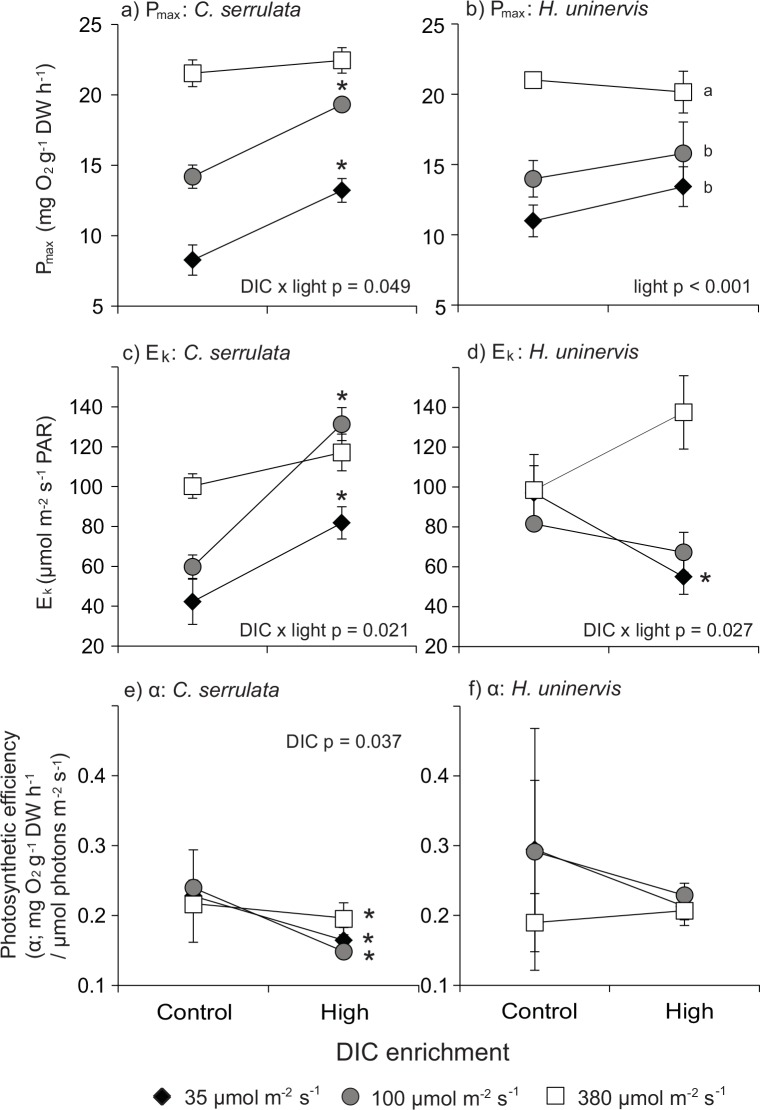
Comparison of photosynthetic parameters between light and DIC for *C*. *serrulata* and *H*. *uninervis* after two weeks exposure. (a-b) P_max_—maximal photosynthetic rate, (c-d) E_k_—saturating irradiances, (e-f) α—photosynthetic efficiency. Letters indicate significant differences between light treatments based on Tukey’s HSD test; asterisks indicate significant differences between DIC treatments based on ANOVA results where there is no interaction between light and DIC, or based on Tukey’s HSD test if an interaction was detected (* p < 0.05). Control DIC = 2063 μM; high DIC = 2215 μM. Means (± S.E.) are given (n = 3).

The increase in maximal photosynthetic rates (P_max_) in *C*. *serrulata* with DIC enrichment depended on light levels (ANOVA: p = 0.049) (Table 2; Fig 2A). The observed increase in P_max_ due to DIC enrichment became smaller with increasing light availability (Fig 2). Post-hoc tests indicated that P_max_ significantly increased with DIC in seagrasses growing at 35 μmol m^-2^ s^-1^ (60% increase) and 100 μmol m^-2^ s^-1^ (36% increase (Tukey HSD: 35 μmol m^-2^ s^-1^: p = 0.014; 100 μmol m^-2^ s^-1^: p = 0.011). There was no significant increase in P_max_ at 380 μmol m^-2^ s^-1^ (p = 0.969). For *H*. *uninervis*, P_max_ increased with light treatments but not with DIC enrichment (ANOVA: light p < 0.001; DIC p = 0.487) (Table 2; Fig 2B). P_max_ was significantly higher at 380 μmol m^-2^s^-1^ than at 35 (71% increase) and 100 μmol m^-2^ s^-1^ (35% increase) (Tukey HSD: 35 μmol m^-2^ s^-1^: p < 0.001; 100 μmol m^-2^s^-1^: p = 0.014).

The response in E_k_ to DIC enrichment was dependent on light levels in *C*. *serrulata*, as shown by a significant light × DIC interaction (ANOVA: p = 0.021) (Table 2; Fig 2C). DIC enrichment resulted in a greater increase in E_k_ at lower light levels (Tukey HSD: 35 μmol m^-2^ s^-1^: p = 0.051; 100 μmol m^-2^ s^-1^: p < 0.001) than at high light levels (Tukey HSD: p = 0.713) (Fig 2). For *H*. *uninvervis*, there was an interactive effect of light and DIC enrichment on E_k_ (ANOVA: p = 0.027) (Table 2; Fig 2D). In low light, DIC enrichment reduced E_k_ by 43% (Tukey HSD: p = 0.028). However, at and above saturating light (100 and 380 μmol m^-2^ s^-1^) DIC enrichment had no significant effect on E_k_.

Compensation irradiance (E_c_) in *C*. *serrulata* varied with light (ANOVA: p = 0.026) but not with DIC enrichment (Table 2). At 380 μmol m^-2^ s^-1^, E_c_ was 102% higher than at 35 μmol m^-2^ s^-1^ (Tukey HSD: p = 0.020), but was not significantly different from that at 100 μmol m^-2^ s^-1^ (Tukey HSD: p = 0.236). In *H*. *uninervis*, there was no main or interactive effect of DIC enrichment and light on E_c_ (ANOVA: p = 0.021; alpha lowered to 0.01) (Table 2).

Photosynthetic efficiency (α) was significantly reduced by DIC enrichment (ANOVA: p = 0.037), but not between light levels (Table 2). No interaction of DIC enrichment and light was detected for α in *C*. *serrulata* (Table 2; Fig 2E). Overall, DIC addition reduced α by 34% for *C*. *serrulata*. For *H*. *uninervis*, there were no main or interactive effects of light and DIC enrichment on α (ANOVA: p = 0.026; alpha lowered to 0.01) (Table 2; Fig 2F).

In summary, both DIC enrichment and light levels influenced the response of photosynthetic parameters in *C*. *serrulata*. The change in parameters such as P_max_ and E_k_ depended on either DIC enrichment or light levels, and also the interaction of both factors. Conversely, photosynthesis in *H*. *uninervis—*P_max_ and E_k_—seemed to be strongly influenced by light treatment but was independent of DIC enrichment.

#### Effects of light levels on HCO3- uptake

**Fig 3:**
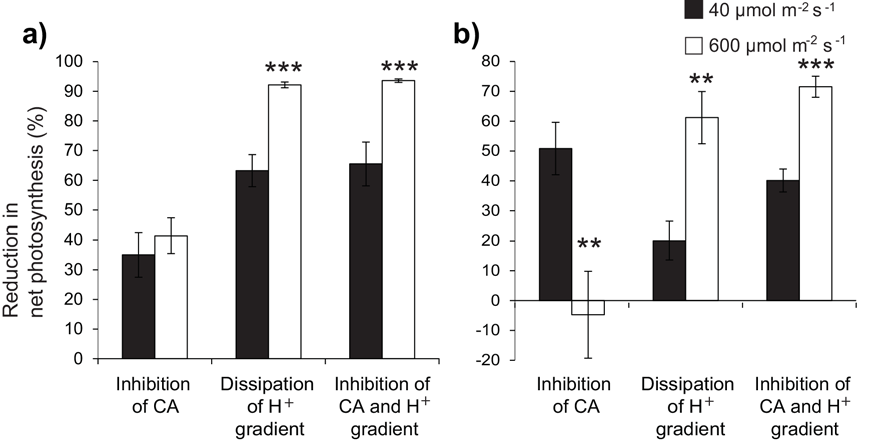
**Reduction in net photosynthetic responses of (a) *C*. *serrulata* and (b) *H*. *uninervis*, when subjected to HCO**_**3**_^**-**^ **uptake inhibitors.** Net photosynthetic responses were expressed relative to control rates in normal seawater. 0.1 mM acetazolamide (inhibition of extracellular CA), 45 mM TRIS buffer at pH 8.0 (dissipation of H^+^ gradient), and a combination of TRIS and acetazolamide (inhibition of CA and H^+^ gradient). Asterisks indicate significant differences between light treatments for each inhibitor or inhibitor combination (** p < 0.01; *** p < 0.001). Means (± S.E) are given (n = 6).

In *C*. *serrulata*, addition of AZ significantly reduced net photosynthesis (paired t-test: t = 4.261, df = 11, p < 0.001) (Fig 3A), indicating that activity of external CA is an important mechanism aiding in HCO_3_^-^ uptake. The reduction in net photosynthesis due to the inhibition of CA-catalysed conversion of HCO_3_^-^ (AZ treatment) was not significantly different between light levels (paired t-test: t = 1.851, df = 5, p = 0.123) (Fig 3A). Addition of TRIS to *C*. *serrulata* also resulted in a strong reduction in net photosynthesis (paired t-test: t = 3.962, df = 11, p = 0.001) (Fig 3A). This suggests that presence of the H^+^ gradient is essential for HCO_3_^-^ transport into the cell. Dissipation of H^+^ gradient alone (TRIS addition) resulted in a higher reduction in net photosynthesis under saturating light (>90%), compared to that under limiting light (60%) (paired t-test: t = 10.126, df = 5, p < 0.001) (Fig 3A). When both CA and H^+^ extrusion were inhibited (addition of both AZ and TRIS), net photosynthesis in *C*. *serrulata* was reduced significantly (paired t-test: t = 4.095, df = 11, p < 0.001) (Fig 3A). Net photosynthesis was reduced to a greater extent under saturating light than under limiting light (paired t-test: t = 7.855, df = 5, p < 0.001) (Fig 3A).

In *H*. *uninervis*, inhibition of extracellular CA (AZ treatment) reduced net photosynthesis by 50% in limiting light (paired t-test: t = -4.188, df = 5, p = 0.009) (Fig 3B), but had no effect in saturating light. This indicated that when light is limiting, CA played a more significant role in HCO_3_^-^ uptake. The dissipation of H^+^ gradient (TRIS addition) decreased net photosynthesis in *H*. *uninervis* (paired t-test: t = 2.755, df = 11, p < 0.001) (Fig 3B). Net photosynthesis decreased more under saturating light conditions (60%) than under low light (20%) (paired t-test: t = 4.380, df = 5, p = 0.007) (Fig 3B). Inhibition of both CA and H^+^ extrusion (addition of both AZ and TRIS) lowered net photosynthesis under both light levels (paired t-test: t = 4.079, df = 11, p < 0.001) (Fig 3B). The extent of inhibition was greater under saturating light than in limiting light (paired t-test: t = 8.315, df = 5, p < 0.001).

## Discussion

Light availability can affect the ability of tropical seagrass to respond to increased DIC provisioning under OA. The present study examined whether *C*. *serrulata* and *H*. *uninervis* were able to adjust growth and photosynthesis responses when exposed to an enriched DIC concentration approximating predicted end-of-century pCO_2_ level (1077ppm) [37] over a range of light levels. Growth of *C*. *serrulata* was stimulated by both DIC enrichment and light availability while growth of *H*. *uninervis* was strongly influenced by light availability only. Interactive effects of DIC enrichment and light treatment were evident in P-E curve parameters in *C*. *serrulata*, while photosynthetic potential in *H*. *uninervis* was affected by the experimental light treatment. The use of CA and H^+^ gradient inhibitors highlighted important differences in carbon uptake mechanisms which may explain some of the differences in responses of the species on a physiological level.

### Growth and net productivity response

The experimental light treatments represented a range of ecologically relevant light conditions that occur in the natural environment. Both *C*. *serrulata* and *H*. *uninervis* were grown in light-saturated conditions under both moderate (100 μmol m^-2^ s^-1^) and high (380 μmol m^-2^ s^-1^) light treatments, where light levels were generally at or above E_k_. The only exception to this was for *C*. *serrulata* at 100 μmol m^-2^ s^-1^ under DIC enrichment, in which E_k_ was 120 μmol m^-2^ s^-1^. In contrast, the lowest light treatment (35 μmol m^-2^ s^-1^) was below E_k_ for both species under all DIC levels, and so photosynthesis was light-limited. All light treatments were above E_c_, and so both seagrass species were in net carbon surplus.

For *C*. *serrulata*, the effect of light and DIC enrichment on growth appeared to be additive, meaning that increasing both DIC and light increased their growth rates. Thus, the highest growth rate was observed at the high light level under DIC enrichment. Further, short term exposure to DIC enrichment meant that plants growing at 35 and 100 μmol m^-2^ s^-1^ were able to grow as fast as plants without DIC enrichment at 100 and 380 μmol m^-2^ s^-1^ respectively, such that DIC enrichment somewhat compensated for lower light availability [19]. While a previous *ex-situ* study showed that growth rates of *C*. *serrulata* did not respond to CO_2_ enrichment [21], our current study showed an increase in growth rates for this species with DIC enrichment, albeit at a very modest 13%. Higher increases in shoot density (194–350%) and above-ground biomass (32–987%) of *C*. *serrulata* were observed in natural CO_2_ vent sites with considerably greater DIC enrichment, compared to adjacent non-CO_2_ enriched sites [51]. Net primary production of *C*. *serrulata* in this study appeared to be strongly limited by light, not DIC concentration. However, previous studies had indicated an increase in net primary productivity with CO_2_ enrichment in this species [21, 51]. Here, the results suggest that light availability plays an upstream role, relative to DIC, in the hierarchical control on seagrass photosynthesis.

Net production and growth in *H*. *uninervis* did not appear to be DIC-limited, as it was less sensitive to an increase in DIC than *C*. *serrulata* under all light treatments. However, previous work has shown that the same population of *H*. *uninervis* can respond to DIC addition by increasing net productivity and growth under similar treatment conditions [21]. Seasonal variation in carbon demand for growth and metabolism might have contributed to the observed differences in response to DIC enrichment between studies [52, 53].

Both DIC enrichment and light availability had been known to stimulate productivity and growth in seagrasses [17, 19, 22, 54]. However, while *C*. *serrulata* increased growth rates with DIC enrichment and light availability, *H*. *uninervis* did not demonstrate a growth response to DIC enrichment. The growth response of *H*. *uninervis* here was limited by light availability, consistent with its net productivity response. Growth responses to DIC enrichment can also be influenced by nutrient availability [55] and water temperature [56]. Sediment pore water nutrients were not measured in the present study and hence it was not possible to assess if overall nutrient availability was limiting seagrass productivity. Knowledge of the interactive effects of environmental factors (light, temperature, nutrients) with DIC enrichment is needed to predict future seagrass productivity responses in the field.

### Photosynthetic potential

Photosynthetic response of *C*. *serrulata* to DIC enrichment depended on treatment light levels. Photosynthetic capacity (P_max_) was higher in *C*. *serrulata* exposed to higher DIC levels for two weeks. *C*. *serrulata* at similar CO_2_ and light conditions to those used here—DIC enrichment and at 400 μmol m^-2^ s^-1^- did increased P_max_ by ~20% [21]. Increases in P_max_ in response to CO_2_ enrichment have also been observed in *Z*. *marina* [19] and *Z*. *noltii* [18]. A larger increase in P_max_ was observed in plants from the limiting (60% increase) compared to the saturating light treatments (0% increase). DIC enrichment can enhance maximum photosynthetic capacity by providing more substrate for fixation and simultaneously lowering photorespiration rates [57]. Light increases maximum photosynthetic capacity by boosting the production of reducing intermediates (e.g. NADPH and ATP) for the carbon reduction cycle [58]. In theory, since both factors have independent mode of actions, their combined effect should be synergistic [59, 60]. The combined sub-additive effect on P_max_ observed in results suggested that the extent of DIC limitation was greater under low light than high light.

Saturating irradiance (E_k_) in *C*. *serrulata* increased with DIC enrichment, with a greater rise in E_k_ observed at lower light levels. Higher saturating light requirements could be driven by the higher photosynthetic capacity due to greater DIC availability, as similarly observed in *Thalassia hemprichii* [17]. The lowering of photosynthetic efficiency with DIC enrichment was unexpected, as increased CO_2_ availability would mean less resources (i.e. ATP) were needed to procure HCO_3_^-^ for photosynthesis [39]. Under CO_2_ enrichment, *Zostera marina* tripled the rate of light-saturated photosynthesis (i.e. P_max_) to reduce the daily photoperiod required for a positive carbon balance [19]. Overall, the studies conducted so far suggest that while DIC enrichment increases the intensity of saturating irradiance required to reach maximum photosynthetic rates, it also reduces the daily period of saturating irradiance required to achieve a net carbon surplus.

Photosynthesis in *H*. *uninervis*, on the other hand, was limited by light, and not by DIC availability. Photosynthetic capacity (P_max_) and E_k_ increased with increasing light levels. Interestingly, increased DIC concentration lowered E_k_ for *H*. *uninervis* shoots growing under low light (35 and 100 μmol m^-2^ s^-1^). This implies that DIC enrichment could, to a certain extent, compensate for low light levels by reducing light requirements in this species. However, the increase in productivity with light availability was higher than the increase with DIC enrichment [61], with maximum photosynthetic rates remaining the highest under high light treatment. Similarly in *Z*. *marina*, CO_2_ enrichment only increased shoot production and below-ground biomass under light-replete but not light-deplete treatments [16].

### Effects of light on DIC utilization

Normally the supply of CO_2_, the preferred DIC species for seagrasses [23, 24], is limited by low free CO_2_ concentration and diffusion rates, and slow conversion from HCO_3_^-^ to CO_2_ [62]. While most seagrasses can utilise HCO_3_^-^ as a DIC source [26, 27] and the concentration of total DIC is non-limiting, the high energetic cost of HCO_3_^-^ uptake makes it a less preferred substrate under low light levels [28, 63]. Light fuels the generation of ATP for both carbon fixation and HCO_3_^-^ uptake [48, 58]. This may explain the apparent paradox that *C*. *serrulata* in our experiment was more DIC-limited at lower light levels than at higher levels. This finding was consistent with the postulation that HCO_3_^-^ utilization is limited at lower light levels [34]. In [34], both deep water (i.e. low light) *Halophila ovalis* and *C*. *serrulata* showed a greater increase in relative electron transport rates (100% and 66% respectively) compared to their intertidal (i.e. high light) counterparts (30% and 20% respectively) when subjected to an 180% increase in DIC concentration. Low light availability can lower HCO_3_^-^ utilization, and OA conditions could boost DIC supply by providing more dissolved CO_2_ [22, 64].

Application of CA and H^+^ gradient inhibitors showed that, in general, both CA and H^+^ gradients are important mechanisms to allow utilisation of HCO_3_^-^ as a carbon source for the two species investigated. This was observed previously in several other seagrass species [22, 28, 38]. For *C*. *serrulata*, the inhibition of carbonic anhydrase (CA) and H^+^ extrusion both reduced net photosynthesis. The strong dependence on H^+^ extrusion for HCO_3_^-^ utilisation in *C*. *serrulata* meant that adequate light levels were needed before HCO_3_^-^ can be used efficiently for photosynthesis. Therefore, at limiting light levels this species probably depends more on CO_2_ diffusion. CA-mediated HCO_3_^-^ dehydration in acidic zones was thought to be a more efficient means of HCO_3_^-^ utilization than CA conversion alone, as the CO_2_ concentration at equilibrium is higher within acidic zones than at normal seawater pH, thus driving the inward diffusion of CO_2_ [28]. Despite this, [26] and [38] demonstrated that extracellular CA-catalysed HCO_3_^-^ conversion to CO_2_ (without H^+^ extrusion sites) was enough to support photosynthetic demand in *C*. *serrulata*. Differences between their findings and ours could be due to long term acclimation of conspecifics to different source meadow environments, or that there were genotypic variation between seagrass populations [65, 66].

*Halodule uninervis* appeared to vary HCO_3_^-^ uptake mechanisms, enabling it to use HCO_3_^-^ over a wider light range for photosynthesis. Under low light conditions, CA-mediated conversion of HCO_3_^-^ contributed substantially to the carbon supply for photosynthesis [28]; when light became saturating, HCO_3_^-^ uptake was supplemented by H^+^ co-transport with HCO_3_^-^. Extracellular CA-mediation of HCO_3_^-^ uptake is less likely to depend on light availability, when compared to the H^+^ extrusion-driven co-transport of HCO_3_^-^ [26, 29]. This flexibility between HCO_3_^-^ utilization pathways suggested *H*. *uninervis* was able to mediate, to a certain extent, DIC limitation under low light conditions. Similarly, efficient use of HCO_3_^-^ utilization mechanisms was thought to account for the lack of photosynthetic responses to DIC enrichment in marine macroalgae [67].

Differential sensitivity to photosynthetic carbon between seagrass species could be due to species variation in DIC utilization mechanisms [38, 64] and extent of carbon-limitation [9, 64]. Species such *C*. *serrulata* (this study) and *Thalassia testudinum* [64] would benefit more from increased CO_2_ supply under OA conditions since they were limited in their ability to utilise the dominant HCO_3_^-^ in seawater. Less DIC limited species, like *H*. *uninervis* (this study), *Halodule wrightii* and *Syringodium filiforme* might show a smaller response to DIC enrichment [64].

### Light and OA as drivers of seagrass productivity

Under future scenarios of OA, marine macrophytes like seagrass could benefit, from both increased DIC concentration and a proportional rise in CO_2_ [15, 25]. While short-term (e.g. this study) and long-term [68] studies have documented varying degrees of DIC limitation in seagrasses, physiological processes that could regulate responses to increased DIC over intermediate time-scales remain unexplored for seagrasses. Epiphytic algae may also benefit from higher CO_2_ [35], and in turn compete with seagrasses for the same resources. Their effects would vary with their composition, biomass and the turnover rates of seagrass leaves [69]. Furthermore, many seagrass habitats are primarily light-limited [8]. The range of light levels used in this experiment, representing the recent light exposure history (~ five years) of the seagrasses at their source meadow, was already much reduced compared to pre-European settlement due to a four-times increase in sediment runoff [70]. Reduced light availability, due to increased sedimentation and epiphytic algal growth, can negate positive OA effects on seagrass growth [35]. Our study showed that the rise in light availability elicited a greater increase in seagrass productivity than DIC enrichment. Compared to OA, variation in water clarity occurs over shorter frequencies and with greater intensities, thus playing a more imminent role in controlling seagrass productivity. Hence, to ensure continued productivity in seagrass meadows in the future, changes in water quality and OA has be to studied and managed in unison.

## Supporting Information

S1 Fig Distribution of daily light sums (photosynthetically active radiation levels) at Cockle Bay, Magnetic Island, Great Barrier Reef during the dry and wet seasons. Distribution of PAR was measured at seagrass canopy height using planar 2π PAR loggers from September 2009 to July 2014.
(EPS)

S2 Fig Light spectra of Aqua Illumination LED Sol at different settings. At 100% blue channel only (blue), 100% white channel only (orange) and 100% both blue and white channels (black). Light spectra were measured using Jazz spectrometer on SpectraSuite software (OceanOptics, USA).
(JPG)

S1 Table Average incubation time, and approximate time range for DIC limitation to occur in 200 mL of ambient seawater during trials, for each light step of P-E curve. Leaf material used during trials was 5–12 mg in dry weight. Light steps in bold indicate a replacement of fresh filtered seawater prior to incubation during actual measurements.
(DOCX)
