# Supplementary figures and images for: Light Levels Affect Carbon Utilisation in Tropical Seagrass under Ocean Acidification
Source: PLoS One. 2016 Mar 3;11(3):e0150352. doi: 10.1371/journal.pone.0150352 (PMC4777487; doi:10.1371/journal.pone.0150352)

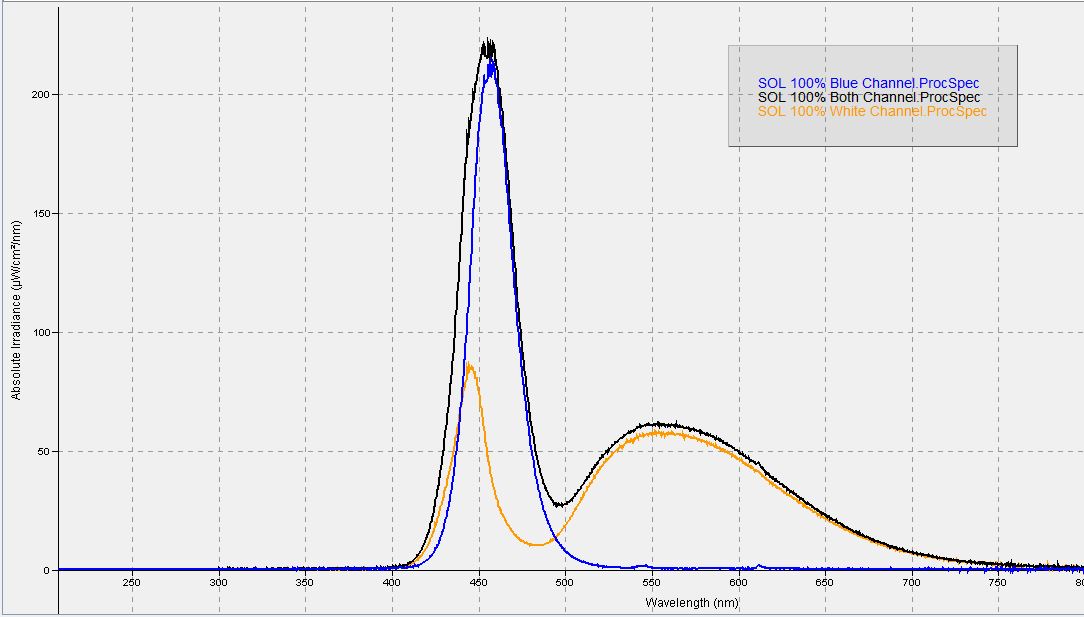

Supplement: S2 Fig — At 100% blue channel only (blue), 100% white channel only (orange) and 100% both blue and white channels (black). Light spectra were measured using Jazz spectrometer on SpectraSuite software (OceanOptics, USA). (JPG) [file pone.0150352.s002.JPG]
