# Supplementary material for: Light Levels Affect Carbon Utilisation in Tropical Seagrass under Ocean Acidification
Source: PLoS One. 2016 Mar 3;11(3):e0150352. doi: 10.1371/journal.pone.0150352 (PMC4777487; doi:10.1371/journal.pone.0150352)
Supplement: S1 Table — Leaf material used during trials was 5–12 mg in dry weight. Light steps in bold indicate a replacement of fresh filtered seawater prior to incubation during actual measurements. (DOCX) [file pone.0150352.s003.docx]

**S1 Table. Average incubation time, and approximate time range for DIC limitation to occur in 200 mL of ambient seawater during trials, for each light step of P-E curve.** Leaf material used during trials was 5 – 12 mg in dry weight. Light steps in bold indicate a replacement of fresh filtered seawater prior to incubation.

| Light step | Average incubation time (min) | Time for DIC limitation (min) |
| --- | --- | --- |
| 10 | 25 | Not measured |
| 20 | 25 | 400 – 480 |
| **35** | 25 | 160 – 170 |
| 70 | 25 | 70 – 80 |
| **100** | 25 | 50 – 55 |
| 200 | 25 | 40 – 50 |
| **380** | 25 | 40 – 50 |
| 520 | 25 | 35 – 40 |
| **600** | 25 | 30 – 50 |
